# Dynamic SST for monitoring position and changes in kyphosis/lordosis angles

**DOI:** 10.1186/1748-7161-8-S2-O32

**Published:** 2013-09-18

**Authors:** Helmut Diers, Christian Diers, Evelyn Firle, Sven Mooshake, Amira Basic, Marco Kleist, Kjell Heitmann

**Affiliations:** 1 DIERS International GmbH, Schlangenbad, Germany

## Background

Spine & Surface Topography (SST) has been used to perform spine diagnostics for more than 20 years. Until recently, static scans were primarily used, as dynamic scans were not properly scientifically and clinically evaluated. Also, the hardware required to perform the dynamic scan was expensive and too slow for a proper clinical use. Due to the emergence of new technology, it is now possible to record and analyze surfaces with a frequency of up to 50 Hertz. The formetric 4D (DIERS International GmbH, Schlangenbad, Germany) static and dynamic SST system records and reconstructs the spinal position of a normally standing patient, with an additional measurement, performs the same tasks while the patient isduring walking in at a sequence of 50 fps.[1] The reconstruction of the spine is done according to follows the methods of Hierholzer / Drerup.[2]

## Purpose

To use static and dynamic surface topography in monitoring changes in kyphosis and lordosis angles during the normal walking stride of a patient (treadmill, 5-10 sec, 50 fps) in comparison to a patient in a normal standing position.

## Methods

Using the formetric 4D, 100 static and dynamic measurements of both pathologic and healthy patients were taken. For this study, the output parameters for the angles ICT-ITL (kyphosis) and ITL-ILS (lordosis) were analyzed.[3] For dynamic measurements the median values for kyphosis and lordosis were used and compared with the values from static measurements using linear regressions.

## Results

The results of linear regressions between the static and dynamic measurements of kyphosis and lordosis angles show a high significance. This validates the usability of dynamic SST compared to static SST. A second result shows that in most of the observed patients the median of kyphosis and lordosis angles from dynamic SST is smaller than in a static SST.

## Conclusions and discussion

Dynamic SST can be used for further studies of kyphosis and lordosis angles. Dynamic SST gives additional information for lordosis and kyphosis angles that cannot be seen in static SST nor in other static measurement technologies.
